# Practical Methods for Vehicle Speed Estimation Using a Microprocessor-Embedded System with AMR Sensors

**DOI:** 10.3390/s18072225

**Published:** 2018-07-10

**Authors:** Vytautas Markevicius, Dangirutis Navikas, Adam Idzkowski, Darius Andriukaitis, Algimantas Valinevicius, Mindaugas Zilys

**Affiliations:** 1 Department of Electronics Engineering, Kaunas University of Technology, Studentu Street 50–418, LT-51368 Kaunas, Lithuania; dangirutis.navikas@ktu.lt (D.N.); darius.andriukaitis@ktu.lt (D.A.); algimantas.valinevicius@ktu.lt (A.V.); mindaugas.zilys@ktu.lt (M.Z.); 2 Faculty of Electrical Engineering, Bialystok University of Technology; Wiejska Street 45D, PL-15351 Bialystok, Poland; a.idzkowski@pb.edu.pl

**Keywords:** magnetic field measurement, magnetic sensors, speed estimation, error analysis, computational complexity

## Abstract

The proper operation of computing resources in a microprocessor-embedded system plays a key role in reducing computing time. Processing the variable amount of collected data in real-time improves the performance of a microprocessor-embedded system. In this regard, a vehicle’s speed measurement system is no exception. The computing time for evaluating any speed value is expected to be reduced as much as possible. Four computational methods, including cross-correlation, are discussed. An exemplary pair of recorded signals presenting the change in magnetic field magnitude is analyzed. The sample delay values are compared. The results of the evaluated speed and the execution time of the program code are presented for each method based on a dataset of 200 randomly driven vehicles. The results of the performed tests confirm that the cross-correlation-based methods are not always reliable in situations when the sample size is small, i.e., it is a segment of the impulse response caused by a driving vehicle.

## 1. Introduction

Time series are used in many domains of applied science and engineering. In recent years, scientists and engineers have been working on vehicle detection systems and vehicle speed estimation using wireless sensor networks (WSNs) [1]. Magnetic sensors, as part of a vehicle’s detection system, are mounted under or on the surface of roadway lanes or roadside. The use of two (or more) anisotropic magnetoresistive (AMR) sensors, which are deployed a short and known distance apart, facilitate the estimation of the speed of the vehicle passing over the AMR sensors. This speed is calculated by finding the delay between two signals, i.e., the time difference. The use of these sensors is an alternative to the inductive loop detectors (ILD) [2].

The error in the estimated speed depends on the characteristics of the used sensors, i.e., a range of measured magnetic induction (it is selectable), operating mode (noise is greater in a low-power mode than in a high-performance mode), and output data rate (it is not greater than 1 kHz) [3].

The way of driving a car over the sensors has an effect on the result. In the ideal case, while a car follows the line of two sensors at a constant speed, the shapes of both signals are the same and their areas under the curves are equal. In reality, it is a rare situation. In addition, environmental conditions, such as temperature changes and background magnetic noise, can cause an additional error.

Regarding a microprocessor-embedded system where computing resources are limited and fast signal processing is important, the accuracy of the estimated speed also depends on the computing method used. This article is focused on the methods selected for computing speed.

## 2. Literature Study

The simplest computing method is based on the detection time of two sensors. It returns a mean absolute percentage error (MAPE) of 10–20% [4,5] in comparison to the results obtained by GPS. However, for estimating speed, the cross-correlation function [4] or normalized cross-correlation function [6] is often used. The time location of a maximum value of cross-correlation function is assumed as a result. Assuming a stationary stochastic process, i.e., constant vehicle speed within the cross-correlation interval [4], this method is accurate and gives results over a wide range of speeds with an error of up to 7% [1,4,5]. However, the disadvantage is that with a high signal sampling, the computational complexity becomes large and many arithmetic operations must be performed to obtain the final result. Assuming the sample size *N* for each signal, the number of these operations is *N*^2^. This limits the possibility of implementing the cross-correlation based algorithm in a real-time small-scale microprocessor-embedded system. The same applies to correlation optimized warping (COW) [7], which is a more effective method at low speeds—when a vehicle stops and starts [8]. Both methods are robust against noise.

One of the ways to reduce the computational complexity is zero padding in a selected segment of the interval from 0 to 2*N* − 1 and the use of DFT (discrete Fourier transform) and IDFT (inverse discrete Fourier transform) [5]. Another way is to use a smaller sample size for cross-correlation. The method is based on finding maximum points in both signals and then performing a computation around those points [6]. As required, the maximum signal delay must be sufficiently less than the sample size. The result depends on the accepted threshold and the sampling frequency of both signals. The precision of the estimated result can be improved by using linear interpolation. In this way, for example, a 10-times greater resolution of the result can be obtained. The error of the estimated speed depends strictly on the sample size.

The algorithm based on computing the sum of absolute differences (SAD) in the function of time-shift (modulus difference processing algorithm) has also been known for a long time [9]. The use of this function provides comparable results to the cross-correlation function in the case of the ultrasonic Doppler signals. In real-time processing, this algorithm is three times faster [9] compared to the algorithm based on the cross-correlation. It contains only adding and subtracting operations, not adding and multiplying. In this method, a minimum of SAD function represents the delay between two signals (estimated speed). A speed value is needed to estimate the length of a vehicle. Therefore, it is important to use the most reliable method of speed estimation, while maintaining the relatively low complexity of its calculation.

Following a literature review, these additional studies have been warranted to verify that the above-mentioned methods can provide analogous results, although their computing times may be very different and sometimes too long (>50 ms). Moreover, it is not clear at which location in the impulse response, caused by a driven vehicle, a speed evaluation process should be started. In the literature, quite a complicated segmentation of magnetic signal magnitude is often proposed [4,10]. Based on the information gathered and the experiments conducted, we present selected techniques for reducing the sample size as much as possible in order to keep the correct result, e.g., within a tolerance of ±2 samples of the evaluated sample delay.

## 3. Vehicle Detection System

**Figure 1:**
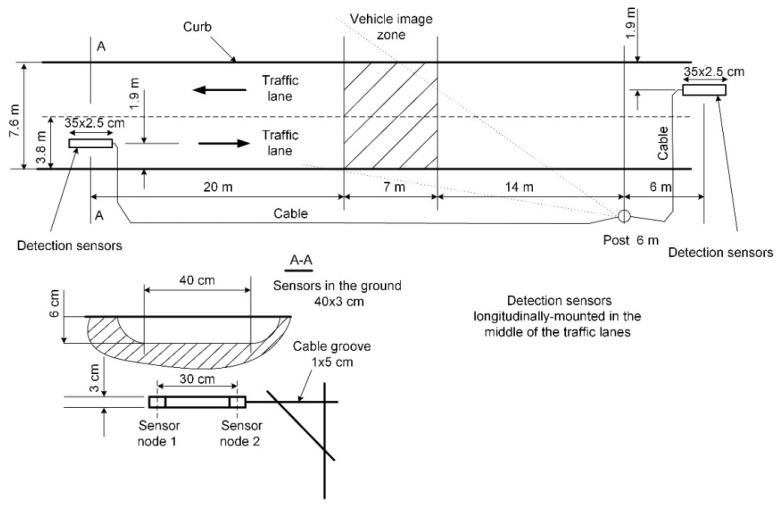
The deployment of detection sensors on the roadway.

**Figure 2:**
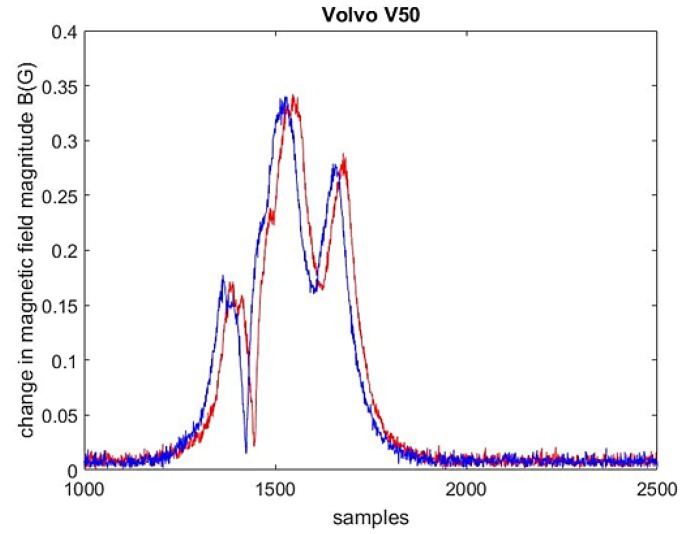
The change in magnetic field magnitude observed in two sensor nodes while a station wagon traveled at 102.9 km/h (sample delay equals 21 samples).

The system is able to record data from two detection zones (Figure 1). Each of them has two nodes (1 and 2) mounted in a flat rectangular-shaped strip of 35 cm × 2.5 cm. Each node contains two three-axis digital magnetic field sensors (LIS3MDL, STMicroelectronics Inc., Santa Clara, CA, USA), FSR = ±4 G (Full Scale Range), 6842 LSB/G. The strips are mounted under the surface of the roadway lanes near the Paluknys district in Lithuania. The 32-bit STM32F401RBT6 microcontroller (STMicroelectronics Inc., Santa Clara, CA, USA) is powered by a 3 V DC supply. The sampling rate of this system is 2 kHz. An SPI interface is used for communication between the microcontroller and the sensors. RAW data from sensor nodes along with the pictures of passing vehicles are recorded on a hard disk and stored in a database. The sensor nodes represent the *x*, *y*, *z* components of the magnetic field induction. The exemplary changes in the magnitude of the magnetic field induction are presented in Figure 2. Data access and video stream are enabled by a website and the Long-Term Evolution (LTE) network.

## 4. Speed Estimation

When used from a static site, radar speed-measuring devices (down-the-road) display, with an accuracy of +2, −3 km/h, the correct speed of a target vehicle that is traveling at 32 to 160 km/h [11]. Recently, a lot of research has been carried out developing them [12,13].

Two longitudinally spaced sensor nodes are required to estimate vehicle speed in the system (Figure 1). The most accurate results possible are obtained by deploying sensors that are further away from each other [5,14]. However, too long of a distance between the sensors involves a risk of signal distortion, which may be caused by a vehicle maneuver. It affects the speed result. This distance is 30 cm in the designed vehicle detection system.

At first, the computations of the vehicle’s speed were performed using four algorithms. Input data relate to 200 vehicles of different types that were driven at speeds from 30 to 150 km/h. The respective sample delay varied from 12 to 60 samples.

### 4.1. Methods and Computational Complexity

Speed was evaluated using four methods:

- method 1—the location of the maximum value of the cross-correlation function in the time domain [15],
- method 2—the location of the minimum value of the SAD function in the time domain [9],
- method 3—the location of the maximum value of the circular convolution of two sequences (using DFT and IDFT algorithms) [5,16],
- method 4—the difference in gravity (mass) centers of two discrete signals [17].

In method 1, the location of the highest peak of the cross-correlation function *f* gives the sample delay Δ*n*, which is recalculated to the time delay Δ*t*_1_. The time delay is given by the formula

(1) $$\Delta t_{1}=\Delta n\cdot t_{s}=\underset{n}{\arg\max}f[n]\cdot t_{s},$$

(2) $$f[n]=\sum_{m=0}^{N-1}B_{1}[m]\cdot B_{2}[m-n],\begin{array}{cc} & -(N-1)<n<N-1 \end{array},$$

where *B*_1_ and *B*_2_ are the magnitudes of the magnetic field signal, *N* is the sample size, *n* is the lag (a delay in samples), Δ*n* is the sample delay, and *t_s_* is the sampling period.

In method 2, the minimum location of the SAD function *g* gives the sample delay, which is recalculated to the time delay Δ*t*_2_:

(3) $$\Delta \;t_{2}=\underset{n}{\arg\min}g\;[n]\cdot t_{s},$$

(4) $$g\;[n]=\sum_{m=0}^{N-1}|B_{1}[m]-B_{2}[m-n]|,\begin{array}{cc} & n=0,1,\ldots ,N-1 \end{array}.$$

The equation used in method 3 enables the calculation of the linear convolution. The IDFT of the element by element product of the DFTs of the two sequences is computed as follows [5]:

(5) $$h[n]=\mathrm{IDFT}\;(\mathrm{DFT}\;({\tilde{z}}_{1}[n])\;\mathrm{DFT}\;({\tilde{z}}_{2}[n])).$$

Using method 3, the time delay is given by

(6) $$\Delta \;t_{3}=\underset{n}{((\arg\max}h\;[n])-(N-1))\cdot t_{s}.$$

In method 4, the centers of mass of two discrete signals *B*_1_(*n*) and *B*_2_(*n*) are located at

(7) $$CoM_{1}=\frac{\sum_{n=1}^{\begin{array}{l} N \end{array}}B_{1}(n)\cdot n}{\sum_{n=`1}^{\begin{array}{l} N \end{array}}B_{1}(n)},\;CoM_{2}=\frac{\sum_{n=1}^{\begin{array}{l} N \end{array}}B_{2}(n)\cdot n}{\sum_{n=`1}^{\begin{array}{l} N \end{array}}B_{2}(n)}.$$

The difference between *CoM*_1_ and *CoM*_2_ gives the sample delay and, after recalculation, the time delay.

Each aforesaid method applied for a speed estimation method is characterized by different computational complexities (Table 1). A low complexity of the calculations, in order to implement it in the embedded system, is the main objective. However, the accuracy of the speed estimation must be assured.

The other method found in the literature is presented in [10]. It is based on the signal matching and four regions selecting from two signal nodes. The presented algorithm is complicated and is not verified in this paper.

### 4.2. Differences between Results When There Is No Filtering and the Sample Size N is Large

**Figure 3:**
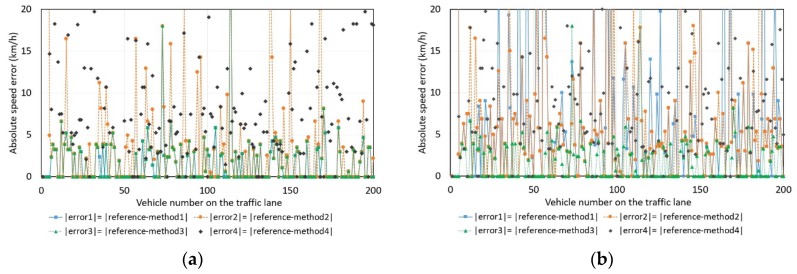
The absolute errors of the estimated speed values by means of methods 1, 2, 3, or 4 with reference to the radar speed readings: (**a**) The magnitude of magnetic field data was used for the calculations; (**b**) *z*-component of magnetic field data was used for the calculations.

**Figure 4:**
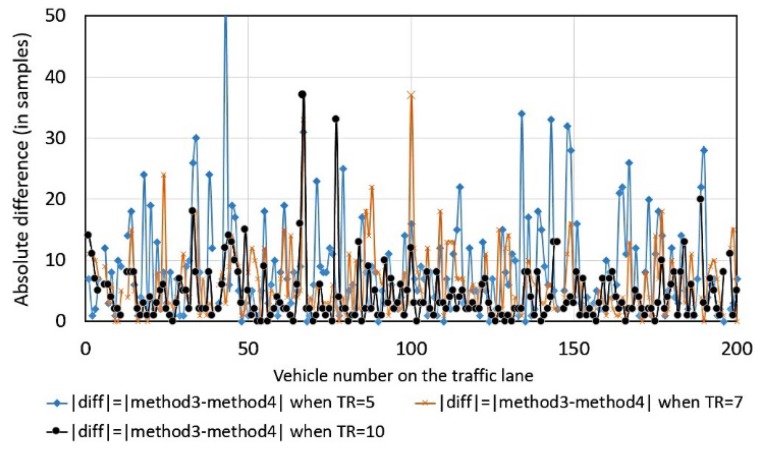
The absolute differences between the estimated value from method 4 with reference to the value from method 3 using *TR* as a parameter, the magnitude of magnetic field data was used for the calculations.

The sample size *N*, required to compute the speed, was constant and equal to 3000 for every driven vehicle. The large sample size means that the input dataset contains more than 100 values of background noise before and after the impulse response caused by a driven vehicle (Figure 2). The results obtained by methods 1, 2, 3, and 4 were compared with the reference radar readings. In Figure 3 and Figure 4, we can see these differences in the results that were made for 200 randomly driven vehicles, i.e., small city cars, sedans, wagons, SUVs, trucks, and buses. By comparing Figure 3a with Figure 3b, we can see that the absolute speed errors are smaller when computations are performed with magnitude data rather than with z-component data.

Methods 1 and 3 provide the same results in 99% of cases when the magnitude-based data were used to compute speed (Figure 3a). The scattering of error in the case of methods 1 and 3 (Figure 3a) is small: the absolute error is less than 3 km/h in 75% of the cases and less than 5 km/h in 94.5% of the cases. The scattering of error in the case of method 2 is higher—less than 3 km/h in 60.5% of cases and less than 5 km/h in 83% of cases.

Method 4 is strongly dependent on the value of threshold, denoted as *TR*, (Figure 4) as opposed to the other methods. The scattering of magnitude-based differences is large: for 40.5% of traveling vehicles, the difference in samples is more than 3; for 28.5%, more than 5 (when threshold *TR* is fixed as *TR*-time mean of background noise).

Methods 1, 2, and 3 are more accurate than method 4. However, they are computationally more complicated.

### 4.3. Differences between Results When Sample Size N Is Small

**Figure 5:**
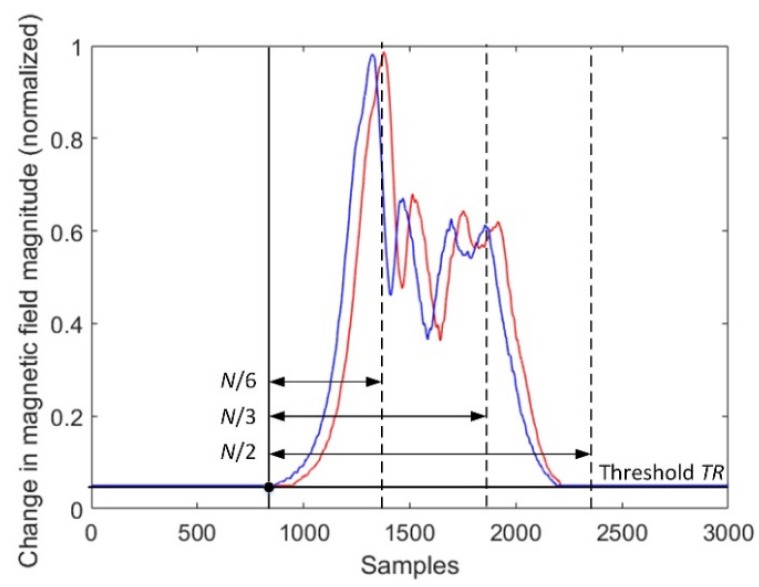
The effect of diminishing the sample size by dividing.

**Figure 6:**
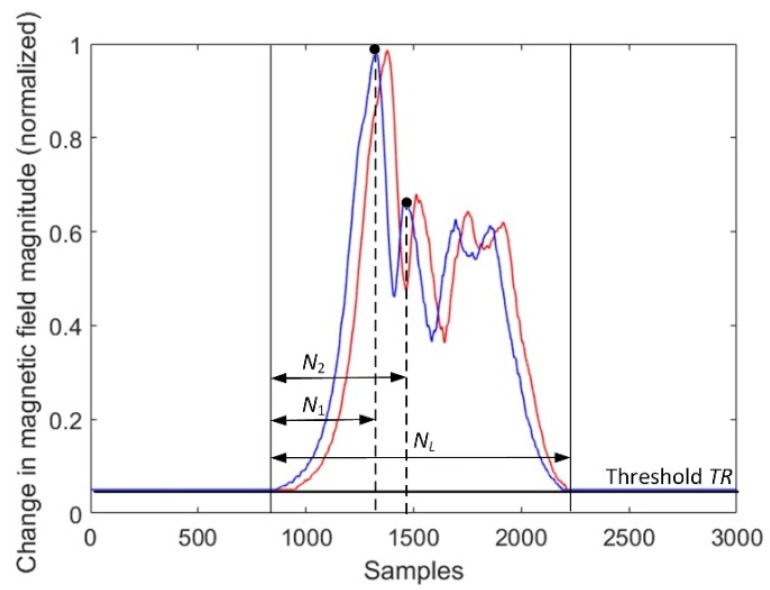
The effect of diminishing the sample size *N* to *N*_2_; *N_L_* is the number of samples above a fixed threshold.

In practical applications, large and constant sample size *N* is unacceptable when using methods 1 and 2 because the execution of the speed calculation function takes too much time (Table 2). Sample size *N* cannot be too large because sometimes the distance between two driven vehicles on the traffic lane is short (trucks can travel even 10 m one after another at speed 90 km/h) and there is a risk of including two vehicles in one speed estimation sample window. The small sample size means that the input dataset contained no background values and only one segment of the impulse response was caused by a driven vehicle (Figure 5 and Figure 6).

In another extreme situation, the speed estimation function has to process a large amount of data if the vehicle is long (e.g., 16.5 m) and travels at 30 km/h. Then, it is necessary to find a method producing similar results with a smaller sample size.

Unfortunately, the cross-correlation-based methods 1 and 3 are not reliable in the situation when the sample size *N* is smaller than the number of samples above a fixed threshold (Figure 5). This results in sample delay (vehicle’s speed) computed on the basis of an uncompleted dataset.

This is visible in Table 2, which presents sample delay estimates for different values of *N*. In cases 4 and 5, methods 1 and 3 provide incorrect results. In this case, the use of method 4 seems to be a better solution.

Table 2 shows that methods 2 and 4 could be effective if we consider using a constant and short *N* continuously for the signals of moving cars in traffic, i.e., in every moment when a change in magnetic field magnitude will cross a given threshold. This motivated the authors to compute the sample delay in another way. In Figure 6, it is shown that two neighboring peaks are localized in the magnetic field magnitude of the first sensor. Then, sample size *N* is equal to *N*_2_, i.e., the distance of the samples from the start to the second significant peak in the signal.

The results obtained for the same vehicle are presented in Table 3. Once more, methods 2 and 4 carry more correct results than methods 1 and 3.

### 4.4. Improvement in Result Resolution

The reason for improving a result resolution is that the error of estimated speeds higher than 90 km/h is large. A linear interpolation function is based on the lowpass interpolation algorithm [18] and it increases the sample rate of the input signal. In Table 4, the speed results are presented for *r* = 10 where *r* is an integer interpolation factor. As presented in Table 4, a 10-times higher resolution is obtained. This results in longer execution times, especially when using methods 1 and 2.

Cases 7 and 9 were tested for 200 vehicles. For the majority of vehicle signatures, method 2 is more accurate than method 4 when *N* < *N_L_*.

**Figure 7:**
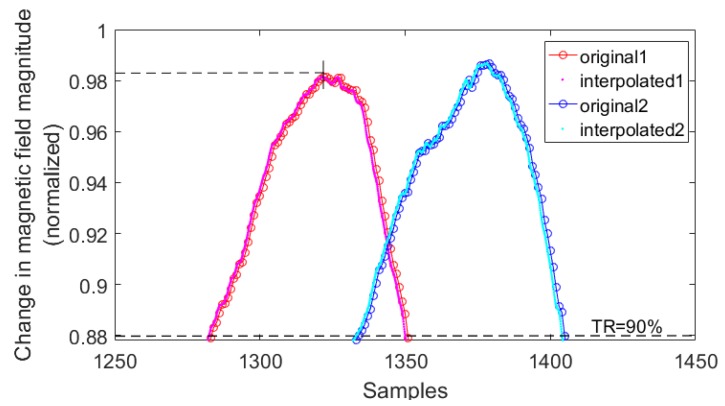
Original signals and signals interpolated with factor *r* = 10.

The final and most effective way to estimate sample delay is to fix the threshold as a high percentage of maximum peak value in the first signal. As a result, the computations are based on the short fragments of interpolated signals. This is illustrated in Figure 7.

Methods 2 and 4 provide incorrect results in cases 10 and 11 (Table 5). Comparing cases 8 and 11, there is little difference in the result values. However, the execution time is about 30 times shorter when using method 1. In case 11, both execution time values are acceptable. For speeds higher than 33.3 km/h, these values do not exceed 60 ms.

Table 6 contains the estimated sample delay for 30 vehicles that traveled within a narrow range of speed—on average, about 100 km/h. The mean absolute difference is taken as a measure of dispersion and it is related to the average sample delay. This percentage difference, calculated from case 6, which is assumed as a reference (no interpolation), and case 8 (with interpolation) is 1.20% (when using method 1) and 1.25% (when using method 3). Changing the threshold from 90% to 80% causes no difference in results when using method 1. In the case of method 3, the percentage differences are 6.72% (calculated from case 6 and case 11) and 7.65% (calculated from case 6 and case 10).

### 4.5. The Influence of Filtering on the Sample Delay Value

**Figure 8:**
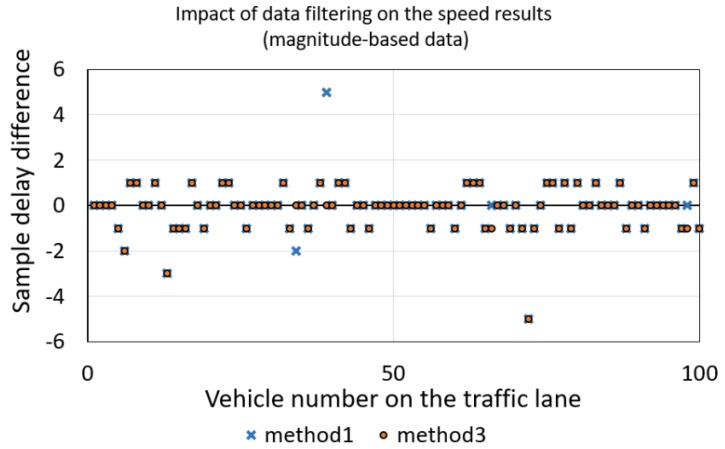
Sample delay difference obtained as a result of data filtering using a ten-sample moving average, *N* = 3000.

Filtering signals by using the moving average is a way of smoothing a signal and finding precisely the points where a signal crosses the threshold. Filtering the magnitude-based data should take a short time and should have no influence on a result. As we can see in Figure 8, a ten-sample moving average (*AVG* = 10) has little impact on the speed estimation result. Considering methods 1 and 3, the result has a difference of about ±1 sample (what is on average ±3 km/h at a speed of 75 km/h) with a probability of 95%.

### 4.6. Importance of Speed Estimation

**Figure 9:**
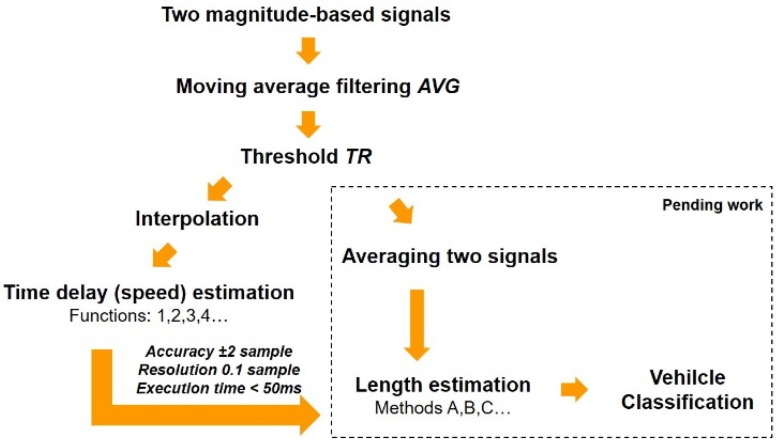
Computation of sample delay as a preliminary task before a vehicle’s length estimation and vehicle classification.

A quick and accurate speed result is needed when a vehicle’s length is calculated in the next step of automated vehicle classification systems (Figure 9).

## 5. Conclusions

Four algorithms and their computational complexity using a microcontroller with two AMR sensors for estimating the speed of a vehicle are presented in this paper. The analysis performed on the signals from three-axis magnetic field sensors mounted on roadways shows that the magnitude-based signals should be processed rather than *z*-component ones. The efficiency of speed estimation methods was tested using a dataset of 200 vehicles. Methods 1 and 3 provided almost the same results when the magnitude-based data were used to compute speed. However, method 3 was 5 to 30 times faster. Methods 1 and 3 were the most accurate; the average absolute error was 1.8 km/h (referring to the radar readings). The same error using methods 2 and 4 was 3.8 km/h and 15.2 km/h, respectively.

Methods 1 and 3 ensured analogous results in 99% of cases when the sample size *N* was large. When the sample size was short *N* << *N_L_* and the signal threshold was 80%, the lower dispersion in a set of data was observed when using method 1 (percentage difference 1.20% vs. 6.72% in comparison to method 3). This means the absolute difference was up to 8 km/h at a speed of 100 km/h. When using method 1, the execution time was 4 times longer. However, for a long vehicle travelling at 33.3 km/h, the execution time of 60 ms is a satisfactory result, considering that it is shorter at higher speeds.

The alternative methods 2 and 4 provided correct results when speed estimation based on a segment of signal (when *N* = *N*_2_) was considered, as presented in Figure 5. In this case, using the cross-correlation methods was not reliable. A sensitivity on the threshold level (it should be relatively high) and a reconstruction of signals by interpolation are the drawbacks of method 4.

The speed estimation result is a compromise between sample size and execution time. Depending on the selected technique (Figure 5, Figure 6 or Figure 7), the SAD method or cross-correlation in time domain is the best choice if it is necessary to handle a small sample size. The use of linear interpolation resulted in achieving a more precise result (Table 6). However, the interpolation is worth being applied if a vehicle travels at speeds higher than 90 km/h.

Method 1, as the most accurate and the slowest, can be applied in the systems based on the high performance microcontrollers, while less accurate and quicker methods 2 or 3 may be implemented in low performance units.

## Figures and Tables

**Table 1:** Computational complexity and output of the selected four methods (*N* = sample size).

Method	Method 1	Method 2	Method 3	Method 4
Computational complexity	*N*^2^ multiplications	*N*^2^ difference operations + *N*^2^/2 modulus operations	*N log*(*N*) DFT operations	2*N* + 2 multiplications + 1 difference operation
Output	Maximum value of 2*N* + 1 elements	Minimum value of *N* + 1 elements	Maximum from *N* + 1 elements	Difference between 2 values

**Table 2:** Estimated sample delay and program execution time for a vehicle that traveled at 33.3 km/h (threshold as a *TR*-time mean of background noise).

Case	Magnitude-Based Signals	Method 1	Method 2	Method 3	Method 4
1	Sample delay when *N* = 3000, *TR* = 0 Execution time (s)	53 0.1188	53 0.1186	53 0.0033	56 0.0037
2	Sample delay when *N* = 3000, *TR* = 10 Execution time (s)	53 0.1130	53 0.1104	53 0.0059	53 0.0030
3	Sample delay when *N*/2, *TR* = 10 Execution time (s)	53 0.0356	53 0.0345	53 0.0069	53 0.0034
4	Sample delay when *N*/3, *TR* = 10 Execution time (s)	34 0.0202	53 0.0199	39 0.0025	53 0.0032
5	Sample delay when *N*/6, *TR* = 10 Execution time (s)	1 0.0098	8 0.0137	500 0.0025	53 0.0034

**Table 3:** Estimated sample delay and program execution time for a vehicle that traveled at 33.3 km/h.

Case	Magnitude-Based Signals	Method 1	Method 2	Method 3	Method 4
6	Sample delay when *N* = *N_L_*, *TR* = 10 Execution time (s)	53 0.0561	53 0.0312	53 0.0033	53 0.0032
7	Sample delay when *N* = *N*_2_, *TR* = 10 Execution time (s)	34 0.0541	53 0.0120	40 0.0026	53 0.0032

**Table 4:** Estimated sample delay and program execution time for a vehicle that traveled at 33.3 km/h; the values were estimated for *r* = 10.

Case	Magnitude-Based Signals	Method 1	Method 2	Method 3	Method 4
8	Sample delay when *N* = *N_L_*, *TR* = 10 Execution time (s)	53.0 1.6974	53.1 1.6963	53.4 0.0067	40.7 0.0050
9	Sample delay when *N* = *N*_2_, *TR* = 10 Execution time (s)	33.9 0.3395	52.8 0.3410	40.1 0.0065	40.7 0.0060

**Table 5:** Estimated sample delay and program execution time for a vehicle that traveled at 33.3 km/h (threshold as a percentage of maximum peak value in the first signal).

Case	Magnitude-Based Signals	Method 1	Method 3
10	Sample delay when *N* << *N_L_*, *TR* = 80% Execution time (s)	53.3 0.0606	52.6 0.0169
11	Sample delay when *N* << *N_L_*, *TR* = 90% Execution time (s)	53.3 0.0591	53.0 0.0133

**Table 6:** Estimated sample delay for vehicles that traveled at speeds from 90 km/h to 112.5 km/h.

Vehicle Number	Case 6 (No Interpolation)		Case 11		Case 10	
	Method 1	Method 3	Method 1	Method 3	Method 1	Method 3
10	16	17	16.2	17.8	16.2	17.6
11	18	18	18.0	17.8	18.0	17.6
13	19	19	19.0	20.9	19.0	21.0
16	19	19	19.3	19.0	19.3	19.0
19	19	19	19.2	21.0	19.2	20.7
26	17	17	17.2	17.5	17.2	18.0
28	19	19	19.0	18.9	19.0	19.0
41	18	18	18.4	19.5	18.4	20.6
56	18	18	18.2	18.4	18.2	18.5
62	17	17	17.2	16.7	17.2	16.7
67	18	18	18.1	18.8	18.1	18.9
76	17	17	17.0	17.1	17.0	16.3
92	16	16	15.9	17.5	15.9	16.8
96	20	20	19.9	21.2	19.9	20.6
103	17	17	17.4	18.7	17.4	18.4
111	14	14	14.3	14.1	14.3	14.1
112	19	19	19.3	20.2	19.3	20.2
123	21	21	20.7	21.5	20.7	21.4
133	19	19	19.3	22.0	19.3	21.4
137	21	21	21.1	18.7	21.1	18.4
142	21	21	21.4	23.5	21.4	23.0
148	17	17	16.8	20.6	16.8	20.2
159	20	20	20.2	24.0	20.2	23.6
163	17	17	16.7	17.3	16.7	16.9
166	15	16	15.5	19.0	15.5	17.1
169	19	19	18.7	18.5	18.7	18.3
174	17	17	17.0	18.6	17.0	17.4
177	17	17	17.4	20.7	17.4	20.5
181	20	20	20.4	21.0	20.4	20.8
184	16	16	15.9	17.0	15.9	16.7
